# CXC Chemokines Exhibit Bactericidal Activity against Multidrug-Resistant Gram-Negative Pathogens

**DOI:** 10.1128/mBio.01549-17

**Published:** 2017-11-14

**Authors:** Matthew A. Crawford, Debra J. Fisher, Lisa M. Leung, Sara Lomonaco, Christine Lascols, Antonio Cannatelli, Tommaso Giani, Gian Maria Rossolini, Yohei Doi, David R. Goodlett, Marc W. Allard, Shashi K. Sharma, Erum Khan, Robert K. Ernst, Molly A. Hughes

**Affiliations:** a Division of Infectious Diseases and International Health, Department of Medicine, University of Virginia, Charlottesville, Virginia, USA; b Department of Microbial Pathogenesis, School of Dentistry, University of Maryland-Baltimore, Baltimore, Maryland, USA; c Center for Food Safety and Applied Nutrition, U.S. Food and Drug Administration, College Park, Maryland, USA; d National Center for Emerging and Zoonotic Infectious Diseases, Centers for Disease Control and Prevention, Atlanta, Georgia, USA; e Department of Medical Biotechnologies, University of Siena, Siena, Italy; f Department of Experimental and Clinical Medicine, University of Florence, Florence, Italy; g Microbiology and Virology Unit, Florence Careggi University Hospital, Florence, Italy; h Division of Infectious Diseases, University of Pittsburgh School of Medicine, Pittsburgh, Pennsylvania, USA; i Department of Pharmaceutical Sciences, School of Pharmacy, University of Maryland—Baltimore, Baltimore, Maryland, USA; j Department of Pathology and Microbiology, Aga Khan University, Karachi, Pakistan; Indiana University Bloomington

**Keywords:** Gram negative, antimicrobial resistance, carbapenem, chemokine, colistin

## Abstract

The continued rise and spread of antimicrobial resistance among bacterial pathogens pose a serious challenge to global health. Countering antimicrobial-resistant pathogens requires a multifaceted effort that includes the discovery of novel therapeutic approaches. Here, we establish the capacity of the human CXC chemokines CXCL9 and CXCL10 to kill multidrug-resistant Gram-negative bacteria, including New Delhi metallo-beta-lactamase-1-producing *Klebsiella pneumoniae* and colistin-resistant members of the family *Enterobacteriaceae* that harbor the mobile colistin resistance protein MCR-1 and thus possess phosphoethanolamine-modified lipid A. Colistin-resistant *K. pneumoniae* isolates affected by genetic mutation of the PmrA/PmrB two-component system, a chromosomally encoded regulator of lipopolysaccharide modification, and containing 4-amino-4-deoxy-l-arabinose-modified lipid A were also found to be susceptible to chemokine-mediated antimicrobial activity. However, loss of PhoP/PhoQ autoregulatory control, caused by disruption of the gene encoding the negative regulator MgrB, limited the bactericidal effects of CXCL9 and CXCL10 in a variable, strain-specific manner. Cumulatively, these findings provide mechanistic insight into chemokine-mediated antimicrobial activity, highlight disparities amongst determinants of colistin resistance, and suggest that chemokine-mediated bactericidal effects merit additional investigation as a therapeutic avenue for treating infections caused by multidrug-resistant pathogens.

## IMPORTANCE

As bacterial pathogens become resistant to multiple antibiotics, the infections they cause become increasingly difficult to treat. Carbapenem antibiotics provide an essential clinical barrier against multidrug-resistant bacteria; however, the dissemination of bacterial enzymes capable of inactivating carbapenems threatens the utility of these important antibiotics. Compounding this concern is the global spread of bacteria invulnerable to colistin, a polymyxin antibiotic considered to be a last line of defense against carbapenem-resistant pathogens. As the effectiveness of existing antibiotics erodes, it is critical to develop innovative antimicrobial therapies. To this end, we demonstrate that the chemokines CXCL9 and CXCL10 kill the most concerning carbapenem- and colistin-resistant pathogens. Our findings provide a unique and timely foundation for therapeutic strategies capable of countering antibiotic-resistant “superbugs.”

## INTRODUCTION

Antimicrobial-resistant bacterial pathogens are a serious threat to global health. Infections caused by multidrug-resistant (MDR) organisms have limited therapeutic options, resulting in frequent treatment failures and increased mortality rates (1). Carbapenem antibiotics have provided a clinical barrier against MDR Gram-negative pathogens; however, the continued emergence and spread of carbapenemases, β-lactamase enzymes capable of hydrolyzing and inactivating carbapenems, imperil the utility of these key antibiotics. Carbapenem-resistant *Enterobacteriaceae* (CRE), including carbapenemase-producing *Klebsiella* spp. and *Escherichia coli*, are resistant to nearly all classes of antibiotics. Consequently, CRE are listed by the U.S. Centers for Disease Control and Prevention as an immediate threat to public health that requires urgent and aggressive action (2).

The challenge posed by CRE has been countered by the use of colistin (CST), a decades-old polymyxin regarded as a last line of defense against highly drug-resistant Gram-negative pathogens. Unfortunately, extensive application of CST in animal husbandry has provided selective pressure for the development of CST resistance. Especially concerning is the dissemination of *mcr*-*1*, a plasmid-borne determinant of polymyxin resistance, among human pathogens (3). CST resistance arising from genetic alterations of chromosomal loci (e.g., *mgrB* and *pmrB*) has also been reported (4); however, unlike *mcr*-*1*, these altered genes are not transmissible between bacterial species. The acquisition of CST resistance by CRE highlights the emergence of extensively drug-resistant “superbugs” that are invulnerable to most, and in some cases all, available antibiotics.

The mounting human and economic burden of MDR bacteria, compounded by steep declines in the development of new antimicrobial agents (2), underscores the importance of developing novel therapeutic strategies. Previous work by our laboratory and others suggests antimicrobial chemokines as one possible avenue to counter the threat posed by antibiotic resistance (5, 6). Indeed, while best known for orchestrating immune cell recruitment to sites of infection, a subset of chemokines has been shown to exert antimicrobial activity against a range of microorganisms (7, 8). Here we report that the CXC chemokines CXCL9 and CXCL10 kill MDR bacterial pathogens, including CRE and CST-resistant clinical isolates.

## RESULTS AND DISCUSSION

### CXC chemokines exhibit bactericidal effects against CRE.

**FIG 1:**
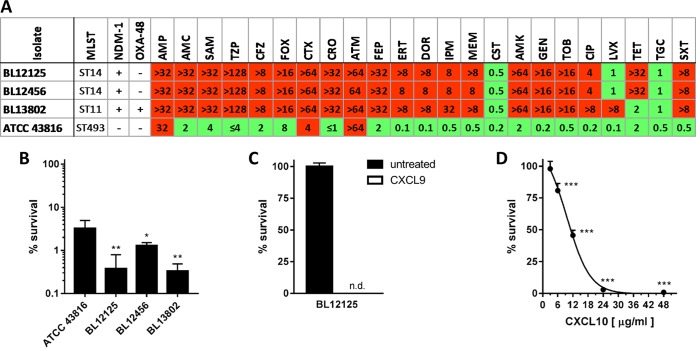
Bactericidal effects of CXC chemokines against CRE. (A) Carbapenem-resistant *K. pneumoniae* isolates and a species-matched control (ATCC 43816) are shown. Multilocus sequence types (MLSTs) and genes encoding the NDM-1 and/or OXA-48 carbapenemases are indicated. Antimicrobial susceptibilities (reported as MIC [µg/ml]) were interpreted as resistant (red) or susceptible (green) in accordance with established breakpoints. The antibiotics tested were ampicillin (AMP), amoxicillin-clavulanic acid (AMC), AMP-sulbactam (SAM), piperacillin-tazobactam (TZP), cefazolin (CFZ), cefoxitin (FOX), cefotaxime (CTX), ceftriaxone (CRO), aztreonam (ATM), cefepime (FEP), ertapenem (ERT), doripenem (DOR), imipenem (IPM), meropenem (MEM), CST, amikacin (AMK), gentamicin (GEN), tobramycin (TOB), ciprofloxacin (CIP), levofloxacin (LVX), tetracycline (TET), tigecycline (TGC), and trimethoprim-sulfamethoxazole (SXT). Bacteria were treated with 48 µg/ml CXCL10 (B) or CXCL9 (C), and survival was measured by CFU determination (limit of detection, 500 CFU/ml; n.d., none detected). Data are expressed as percentages of the respective untreated-control value and represent the mean ± the standard error of the mean (*n* = 3). *, *P* < 0.05; **, *P* < 0.01 (compared to ATCC 43816). (D) CRE isolate BL12125 was treated with increasing concentrations of CXCL10. Data are expressed as percentages of the untreated-control value and represent the mean ± the standard error of the mean (*n* = 3). ***, *P* < 0.001 (compared to the untreated control).

The capacity of CXC chemokines to directly kill CRE was examined by using clinical isolates of MDR *Klebsiella pneumoniae* that possess either New Delhi metallo-β-lactamase-1 (NDM-1) or both NDM-1 and oxacillinase-48 (OXA-48) carbapenemases (Fig. 1A). NDM-1 presents a considerable challenge to antimicrobial therapy because of its high enzymatic and genetic stability (9, 10) and exceptional horizontal mobility among Gram-negative bacterial species (11). On the basis of prior investigations (12), CRE were treated with 48 µg/ml CXCL10. Viability determinations (CFU/ml) demonstrated that CXCL10 killed all of the MDR carbapenemase-producing organisms tested (Fig. 1B). Indeed, CXCL10-mediated killing of CRE was greater than that of the carbapenemase-negative control *K. pneumoniae* ATCC 43816. Human CXCL9 also efficiently killed CRE (Fig. 1C).

Chemokine-mediated antimicrobial activity against CRE was dependent on the chemokine concentration used (Fig. 1D). The half-maximal effective concentration (EC_50_) of CXCL10 for isolate BL12125 was 9.6 ± 1.1 µg/ml (~1.1 µM). This value is comparable to the previously determined bactericidal capacity of human CXCL10 against *Bacillus anthracis* bacilli (EC_50_, 0.5 µM) and *E. coli* (EC_50_, 0.3 to 1.0 µM) and is also similar to those reported for the killing of *K. pneumoniae* by human (EC_50_, 0.3 µM) and murine (EC_50_, 1.6 µM) CCL28 (5, 12, 13). Our findings demonstrate that CXCL9 and CXCL10 kill carbapenem-resistant bacterial isolates that also maintain broad co-resistance against additional classes of antibiotics. Given the increasingly limited options available to treat infections caused by MDR bacterial pathogens, this novel observation highlights the potential utility of chemokine-mediated antimicrobial activity as a foundation for developing innovative therapeutic strategies to counter a range of antibiotic-resistant pathogens.

### CXC chemokines mediate antimicrobial activity against *mcr*-*1*-harboring bacteria.

CST is a cationic lipopeptide that targets Gram-negative bacteria through electrostatic interactions with phosphate groups on the lipid A component of lipopolysaccharide (14). While the genetic basis of CST resistance is diverse, the principal mechanisms are analogous; chemical modification of lipid A phosphates mask their negative charge, thereby decreasing affinity between CST and the bacterial cell envelope (4). Most concerning among determinants of CST resistance is *mcr*-*1*, a horizontally transmissible, plasmid-borne gene that encodes a phosphoethanolamine (pEtN) transferase that catalyzes the addition of pEtN onto phosphate moieties of lipid A (3). MCR-1 has been shown to reduce CST susceptibility in a number of important human pathogens, including *E. coli*, *K. pneumoniae*, *Pseudomonas aeruginosa*, and *Acinetobacter baumannii* (15).

**FIG 2:**
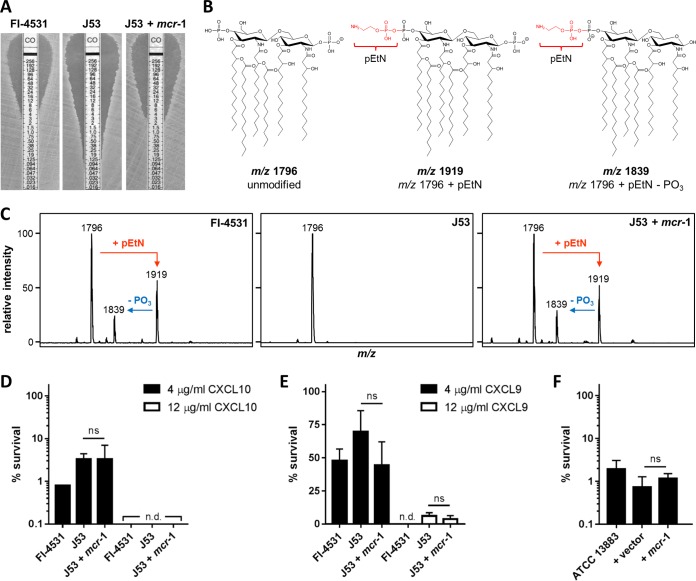
Chemokine-mediated antimicrobial activity against *mcr*-*1*^+^ bacteria. (A) CST susceptibility testing of *E. coli* by modified Etest. CST concentrations are in µg/ml. Images are representative of two or three separate tests. Printed CO indicates a CST test strip. (B) Molecular structures and mass-to-charge (*m*/*z*) ratios of signature ions identified in *E. coli* mass spectra. (C) Lipid extracts were analyzed by MALDI-TOF mass spectrometry. The unmodified base peak (*m*/*z* 1,796) was observed in all spectra. Major ions at *m/z* 1,919 and 1,839, corresponding to pEtN-modified lipid A and pEtN-modified lipid A lacking the second phosphate moiety, were detected only in spectra generated from *mcr*-*1*^+^ isolates. *E. coli* isolates were treated with 4 or 12 µg/ml CXCL10 (D) or CXCL9 (E); *K. pneumoniae* strains were treated with 48 µg/ml CXCL10 (F). Different chemokine concentrations were assayed on the basis of the inherent susceptibilities of these organisms. Survival was measured by CFU determination (limit of detection, 500 CFU/ml; n.d., none detected). Data are expressed as percentages of the respective untreated-control value and represent the mean ± the standard error of the mean (*n* = 3). ns, not significant.

To define the capacity of CXCL9 and CXCL10 to kill *mcr*-*1*^+^ CST-resistant bacteria, three *E. coli* isolates were used, FI-4531, an *mcr*-*1*^+^ clinical isolate; J53, a laboratory reference strain; and J53 *mcr*-*1*, a J53 transconjugant carrying the *mcr*-*1*-containing plasmid from FI-4531. The CST resistance of *mcr*-*1*^+^ isolates was confirmed using a modified Etest (Fig. 2A). To verify the presence or absence of pEtN-modified lipid A in the outer membrane of the above bacterial strains (Fig. 2B; see Fig. S1 in the supplemental material), lipid extracts from each were prepared and analyzed by matrix-assisted laser desorption/ionization time-of-flight (MALDI-TOF) mass spectrometry (Fig. 2C). The canonical, unmodified bis-phosphorylated hexa-acylated base peak was observed in all mass spectra and was the only major ion detected for J53. In addition to the base peak, both FI-4531 and J53 *mcr*-*1* displayed major ions corresponding to pEtN-modified lipid A. Thus, the CST resistance of *mcr*-*1*^+^ strains was appropriately associated with pEtN-modified lipid A.

10.1128/mBio.01549-17.1 FIG S1  Lipid A structures. The molecular structures and *m/z* ratios of major ions identified in *E. coli* and *K. pneumoniae* lipid A mass spectra are shown. Download FIG S1, TIF file, 0.8 MB. Copyright © 2017 Crawford et al. 2017 Crawford et al. This content is distributed under the terms of the Creative Commons Attribution 4.0 International license.

Exposure of FI-4531, J53, or J53 *mcr*-*1* to 4.0 µg/ml human CXCL10 resulted in approximately 2-log killing, as measured by CFU determination, without regard to *mcr*-*1* status (Fig. 2D). Treatment with 12.0 µg/ml CXCL10 consistently resulted in the death of the initial inoculum of each *E. coli* strain below the level of detection (500 CFU/ml). CXCL9 was also observed to mediate similar, marked bactericidal effects against the *E. coli* strains examined (Fig. 2E). Furthermore, introduction of a *mcr*-*1*-carrying recombinant plasmid into CST-susceptible *K. pneumoniae* ATCC 13883 did not promote resistance to chemokine-mediated antimicrobial activity (Fig. 2F), despite conferring CST resistance (MIC, 32 µg/ml) (15). These data demonstrate that CXCL9 and CXCL10 are able to effectively kill *mcr*-*1*^+^ CST-resistant pathogens that maintain pEtN-modified lipid A in their outer membrane.

### Disparate chromosomal determinants of CST resistance differentially impact the bactericidal activity of CXC chemokines.

Similar to pEtN, the modification of lipid A with 4-amino-4-deoxy-l-arabinose (l-Ara4N) reduces the net charge of the bacterial cell envelope, thereby promoting CST resistance. The PmrA/PmrB two-component regulatory system governs the expression of chromosomal loci that encode the enzymes responsible for l-Ara4N synthesis and incorporation into lipid A (16). Discrete, nonsynonymous mutations in *pmrA* or *pmrB* are associated with the constitutive activation of this signaling pathway and CST resistance (4). Particular mutations in PhoP/PhoQ, an upstream activator of the response regulator PmrA (17), and genetic disruption of MgrB, a negative regulator of PhoP/PhoQ signaling (18), also result in the activation of PmrA and CST-resistant phenotypes (4, 19). While not horizontally transmissible, these determinants have been identified in a number of human pathogens and present an emergent clinical challenge (19).

**FIG 3:**
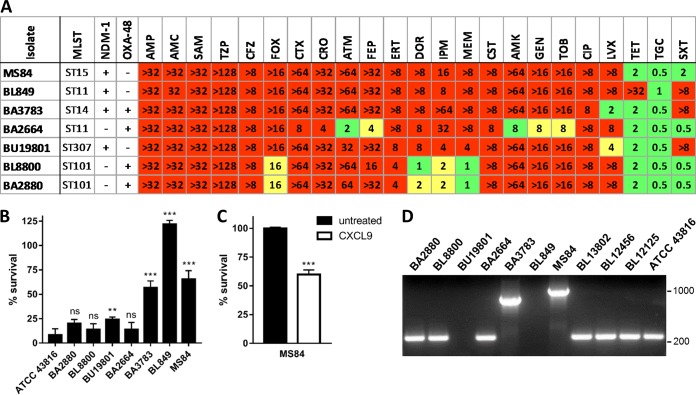
Effects of chromosomal determinants of CST resistance on the bactericidal activity of CXC chemokines. (A) MLSTs, carbapenemase genes, and antimicrobial susceptibilities of CST-resistant *K. pneumoniae* isolates are shown. MICs [µg/ml] were interpreted as resistant (red), intermediate (yellow), or susceptible (green) on the basis of established breakpoints. For definitions of abbreviations, see the legend to Fig. 1. Bacteria were treated with 48 µg/ml CXCL10 (B) or CXCL9 (C), and survival was measured by CFU determination. Data are expressed as percentages of the respective untreated-control value and represent the mean ± the standard error of the mean (*n* = 3). **, *P* < 0.01; ***, *P* < 0.001; ns, not significant (compared to ATCC 43816 [panel B] or the untreated control [panel C]). (D) Visualization of *mgrB* amplicons generated by PCR from the isolates indicated. Amplicon size of intact *K. pneumoniae mgrB*, 235 bp. Markers indicate 1,000 and 200 bp.

To test the effects of chromosomal determinants of CST resistance on chemokine-mediated antimicrobial activity, we examined MDR, *mcr*-*1* negative, CST-resistant *K. pneumoniae* clinical isolates (Fig. 3A). Bacteria were treated with 48 µg/ml CXCL10, and survival was measured by CFU determination (Fig. 3B). Curiously, CXCL10 exhibited strain-specific bactericidal activity against the isolates examined, i.e., three susceptible isolates (<25% survival; BA2664, BL8800, and BA2880), three intermediate isolates (25 to 75% survival; MS84, BA3783, and BU19801), and one unaffected isolate (BL849). The intermediate phenotype displayed by isolate MS84 was also observed upon treatment with CXCL9 (Fig. 3C). MALDI-TOF analysis demonstrated that all of the bacterial isolates examined possessed similar relative amounts of l-Ara4N-modified lipid A in their outer membrane (Fig. S2); thus, the reduction in anionic surface charge afforded by l-Ara4N modification of lipid A does not appear to explain the differences in susceptibility to chemokine-mediated antimicrobial activity. This observation is consistent with our finding that pEtN modification of lipid A also does not reduce susceptibility to CXCL9 or CXCL10. Taken together, these results support the notion that CXCL9 and CXCL10 kill bacterial pathogens via a mechanism distinct, at least in part, from those reliant upon electrostatic interaction as proposed for a number of cationic antimicrobial peptides, including CST.

10.1128/mBio.01549-17.2 FIG S2  *K. pneumoniae* lipid A mass spectra. Lipid A mass spectra obtained from the *K. pneumoniae* isolates examined in this study are shown. CST and CXCL10 susceptibilities are indicated as susceptible (s) or resistant (r). Download FIG S2, TIF file, 0.7 MB. Copyright © 2017 Crawford et al. 2017 Crawford et al. This content is distributed under the terms of the Creative Commons Attribution 4.0 International license.

We next examined the possibility that disparate chromosomal determinants conferring CST resistance account for the noted variability in CXCL10 susceptibility. To identify the specific mutations/disruptions that underlie CST resistance in the above clinical isolates, five loci (*mgrB*, *pmrA*, *pmrB*, *phoP*, and *phoQ*) previously reported to be associated with CST-resistant phenotypes were examined by amplicon sequencing and analysis of whole-genome sequence data (4, 20). All of our CST-resistant, CXCL10-susceptible *K. pneumoniae* isolates (BA2664, BL8800, and BA2880) possessed a nonsynonymous *pmrB* point mutation resulting in a Thr157Pro amino acid substitution. This particular substitution has been proposed to impact dimerization and phosphotransferase by PmrB, leading to constitutive activation of PmrA and, consequently, CST resistance (21). In contrast, CST-resistant organisms that exhibited partial or complete resistance to CXCL10 were found to possess *mgrB* loci disrupted by insertion sequences (MS84, IS*5*-like element; BA3783, IS*1*-like element; BU19801, IS*Kpn25* element) or to be missing the *mgrB* gene entirely (BL849) (Fig. 3D). Functional inactivation of *mgrB* eliminates the negative feedback loop governing PhoP/PhoQ signaling, resulting in both direct and indirect (via PmrD-dependent activation of PmrA) transcriptional activation of the biosynthetic machinery necessary for l-Ara4N modification of lipid A in *K. pneumoniae* (4, 22). No isolates were found to have genetic aberrations previously associated with CST resistance in the *pmrA*, *phoP*, or *phoQ* loci.

That impairment of PhoP/PhoQ autoregulatory control curtails the antimicrobial activity of CXCL10, but direct disruption of PmrA/PmrB does not, reasonably suggests that disparate chromosomal determinants of CST resistance inequitably affect CXCL10 susceptibility. Indeed, while constitutive activation of PmrA, either directly or indirectly, would be expected to result in the incorporation of l-Ara4N-modified lipid A into the bacterial outer membrane, disruption of PhoP/PhoQ autoregulation would also be expected to influence the expression and/or activity of a number of additional enzymes and regulatory systems central to defensive alteration of the bacterial outer membrane (23). These findings highlight the underappreciated prospect that distinct chromosomal determinants of CST resistance differentially impact outer membrane physiology and thus variably affect microbial traits beyond CST resistance.

### Restoration of PhoP/PhoQ autoregulatory control abolishes protection from CXCL10.

To substantiate that *mgrB* disruption and the resultant loss of PhoP/PhoQ autoregulation account for reduced CXCL10 susceptibility, we performed genetic complementation and susceptibility determination. For these experiments, native *K. pneumoniae mgrB* was introduced into isolate MS84. This MDR isolate was chosen for *mgrB* complementation since it demonstrated reduced CXCL10 susceptibility yet was still susceptible to TET and thus amenable to screening selection.

**FIG 4:**
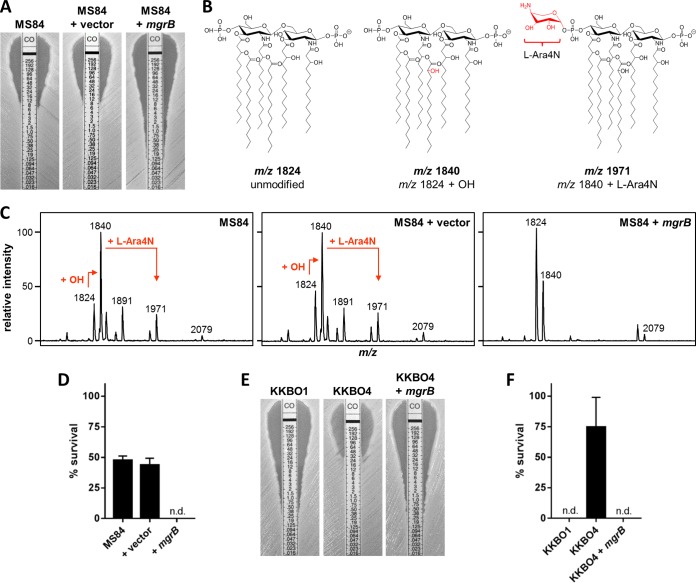
Effect of *mgrB* complementation on resistance phenotypes. (A) CST susceptibility testing of *K. pneumoniae* strains by modified E test. CST concentrations are in µg/ml. Images are representative of three separate tests. (B) Molecular structures and *m/z* values of selected ions identified in *K. pneumoniae* mass spectra. (C) MALDI-TOF analysis demonstrated the canonical *K. pneumoniae* lipid A base peak (*m*/*z* 1,824). Major ions at 1,955 (*m/z* 1,824 + l-Ara4N), 1,971 (*m/z* 1,840 + l-Ara4N), and 1,891 (*m*/*z* 1,971 − PO_3_) were observed in mass spectra from strains harboring disrupted *mgrB*. Additional ions observed at *m/z* 1,840, 2,063, and 2,079 are indicative of lipid A hydroxylation, palmitoylation, or both, respectively. (D) Bacteria were treated with 48 µg/ml CXCL10. Survival was measured by CFU counting (limit of detection, 500 CFU/ml; n.d., none detected). Data are expressed as percentages of the respective untreated-control value and represent the mean ± the standard error of the mean (*n* = 3). (E) CST susceptibility testing by modified E test. Images are representative of two or three separate tests. (F) Bacteria were treated with 48 µg/ml CXCL10. Survival was measured by CFU counting (limit of detection, 500 CFU/ml; n.d., none detected). Data are expressed as percentages of the respective untreated control and represent the mean ± the standard error of the mean (*n* = 3).

Complementation of isolate MS84 with pACYC::*mgrB* restored CST susceptibility (Fig. 4A), elicited the loss of l-Ara4N-modified lipid A (Fig. 4B and C), and markedly increased susceptibility to CXCL10 (Fig. 4D). In contrast, the empty-vector control was equivalent to the MS84 parent strain in all of the regards examined. These data were validated by using an additional set of three *K. pneumoniae* isolates, KKBO1, KKBO4, and KKBO4 *mgrB*. KKBO1 and KKBO4 are sequentially collected, *K. pneumoniae* carbapenemase (KPC)-producing clinical isolates that are CST susceptible or resistant, respectively. The CST resistance of KKBO4 has previously been shown to be caused by insertional inactivation of *mgrB* (24). Genetic complementation with pACYC::*mgrB* reestablished CST susceptibility in KKBO4 (Fig. 4E) and eliminated l-Ara4N-modified lipid A (Fig. S2). Determination of chemokine susceptibility demonstrated that only *K. pneumoniae* isolates harboring intact *mgrB* alleles were fully sensitive to the bactericidal effects of CXCL10 (Fig. 4F). These observations demonstrate that diminished CXCL10 susceptibility is associated with the functional inactivation of *mgrB*.

A pair of additional observations can be made from the lipid A mass spectra presented in this work. In *K. pneumoniae*, the PhoP/PhoQ-activated loci *lpxO* and *pagP* encode lipid A-modifying enzymes responsible for the hydroxylation of 2′-linked myristate (LpxO) and the addition of palmitate (C16:0) onto the hydroxyl group of hydroxymyristate (PagP) (25). The reinforcement of hydrogen bond networks by hydroxylated lipid A and the strengthening of hydrophobic interactions by palmitoylated lipid A are proposed to fortify outer membrane barrier function and impede penetration by antimicrobial peptides. Although hydroxylation and palmitoylation of lipid A have each been reported to promote bacterial resistance to antimicrobial peptides (26, 27), the presence of these PhoP/PhoQ-regulated modifications did not appear to account for CXCL10 resistance in isolates harboring disruptions in *mgrB*. This conclusion is evidenced by the detection of 2-hydroxylated lipid A (12/12 isolates) and palmitoylated lipid A (6/12 isolates) in both CXCL10-susceptible and -resistant *K. pneumoniae* strains (Fig. 4; Fig. S2).

Further investigation is required to clarify the molecular basis of PhoP/PhoQ-dependent protection from the bactericidal effects of CXCL9 and CXCL10 and to determine whether this limitation can be overcome. Of interest, *K. pneumoniae* isolate BU19801 demonstrated *mgrB* disruption yet was only marginally resistant to CXCL10 (Fig. 3). This phenotype is distinct from the pronounced CXCL10 resistance exhibited by other clinical isolates harboring interrupted *mgrB* and may provide an opportunity to elucidate the molecular basis of protection from CXCL10.

While it is unknown whether antimicrobial chemokines act through a common mechanism, they are generally considered to target bacteria via electrostatic interactions between positively charged topological “patches” present on the chemokine and negatively charged constituents of the bacterial cell envelope, notably, the lipid A component of lipopolysaccharide in Gram-negative bacteria (7, 28). This basic mechanism is analogous to those proposed for a number of cationic antimicrobial peptides, including CST, and is in agreement with many experimental results (6, 29). However, pEtN or l-Ara4N modification of lipid A phosphate moieties and consequent reduction of the bacterial surface charge were not found to specifically diminish the bactericidal effects of CXCL9 and CXCL10. These findings suggest that factors other than charge-based interactions may be critical for the bactericidal action of these CXC chemokines. This notion is supported by previous findings that CXCL10 may interact directly with the conserved bacterial component FtsX, which displays homology with CXCR3, the cognate cellular receptor of CXCL10 (30, 31). Elucidation of the underlying principles of chemokine-mediated antimicrobial activity will inform the development of innovative strategies for treating infections caused by antimicrobial-resistant pathogens.

## MATERIALS AND METHODS

### Bacterial strains and culture conditions.

The bacterial isolates used in this study and their derivatives are summarized in Table 1. MDR *K. pneumoniae* clinical isolates BL849 (CFSAN044563), BU19801 (CFSAN044564), MS84 (CFSAN044565), BL12125 (CFSAN044566), BL12456 (CFSAN044568), BA3783 (CFSAN044569), BL13802 (CFSAN044570), BA2664 (CFSAN044571), BL8800 (CFSAN044572), and BA2880 (CFSAN044573) were obtained in Pakistan (20). *K. pneumoniae* ATCC 43816 was used as a species-matched control for phenotypic assays. Sequence typing of the above isolates was performed and assignments were made in accordance with the Institut Pasteur *K. pneumoniae* multilocus sequence typing scheme (http://bigsdb.pasteur.fr/). *K. pneumoniae* KKBO1 and KKBO4 (24), and *E. coli* FI-4531 were obtained in Italy (32). *E. coli* J53 and *K. pneumoniae* ATCC 13883 were used as host backgrounds for *mcr*-*1*-containing plasmids. *E. coli* DH5α was used as an intermediate in molecular cloning. Positive control strains for antimicrobial susceptibility testing included *Staphylococcus aureus* ATCC 29213, *Enterococcus faecalis* ATCC 29212, *E. coli* ATCC 25922, and *P. aeruginosa* ATCC 27853. Unless otherwise noted, bacteria were cultured in Luria-Bertani (LB) medium at 37°C with continuous shaking. *E. coli* FI-4531 and J53 *mcr*-*1* were grown in the presence of 1 µg/ml CST freshly prepared. *K. pneumoniae* MS84 derivatives and KKBO4 *mgrB*, each harboring pACYC constructs, were grown in the presence of 25 µg/ml TET. *K. pneumoniae* strains derived from ATCC 13883 and containing pBCSK constructs were grown in the presence of 50 µg/ml chloramphenicol.

**TABLE 1:** Bacterial isolates used in this study

Species and isolate	Relevant trait(s)	Source
*E. coli*		
FI-4531	*mcr*-*1*^+^, CST^r^	32
J53	Reference/recipient, CST^s^	38
J53 *mcr*-*1*	*mcr*-*1*^+^ transconjugant, CST^r^	This study
*K. pneumoniae*		
BL12125	MDR, NDM-1^+^, CST^s^	20
BL12456	MDR, NDM-1^+^, CST^s^	20
BL13802	MDR, NDM-1^+^, OXA-48^+^, CST^s^	20
ATCC 13883	Reference/recipient, CST^s^	ATCC^a^
ATCC 13883 + vector	pBCSK, CST^s^	15
ATCC 13883 *mcr*-*1*	pBCSK::*mcr*-*1*, CST^r^	15
MS84	MDR, NDM-1^+^, CST^r^ (*mgrB* disruption)	20
MS84 + vector	pACYC, CST^r^	This study
MS84 *mgrB*	pACYC::*mgrB*, CST^s^	This study
BL849	MDR, NDM-1^+^, CST^r^ (*mgrB* disruption)	20
BA3783	MDR, NDM-1^+^, OXA-48^+^, CST^r^ (*mgrB* disruption)	20
BA2664	MDR, OXA-48^+^, CST^r^ (*pmrB* Thr157Pro)	20
BU19801	MDR, NDM-1^+^, CST^r^ (*mgrB* disruption)	20
BL8800	MDR, OXA-48^+^, CST^r^ (*pmrB* Thr157Pro)	20
BA2880	MDR, OXA-48^+^, CST^r^ (*pmrB* Thr157Pro)	20
KKBO1	MDR, KPC^+^, CST^s^	24
KKBO4	MDR, KPC^+^, CST^r^ (*mgrB* disruption)	24
KKBO4 *mgrB*	pACYC::*mgrB*, CST^s^	24

^a^ ATCC, American Type Culture Collection, Manassas, VA, USA.

### Antimicrobial susceptibility testing and carbapenemase gene detection.

Antimicrobial resistance was first established with the Vitek 2 system (BioMérieux, Marcy l’Etoile, France). Broth microdilution, in accordance with Clinical and Laboratory Standards Institute (CLSI) guidelines (33), was subsequently used to determine the MICs of antibiotics representing the main families of antimicrobial agents, i.e., ampicillin, amoxicillin-clavulanic acid, AMP-sulbactam, piperacillin-tazobactam, cefazolin, cefoxitin, cefotaxime, ceftriaxone, aztreonam, cefepime, ertapenem, doripenem, imipenem, meropenem, CST, amikacin, gentamicin, tobramycin, ciprofloxacin, levofloxacin, TET, TGC, and trimethoprim-sulfamethoxazole. Antimicrobial susceptibilities were interpreted on the basis of 2016 CLSI breakpoints (34), except those for CST and TGC, which are not defined by the CLSI. The European Committee for Antimicrobial Susceptibility Testing (EUCAST) breakpoints were used for CST and TGC (35). The presence of genes encoding the NDM-1 and OXA-48 carbapenemases was determined by PCR amplification (36).

### CST Etest.

While CST resistance was established by broth microdilution, the maintenance of resistant phenotypes was confirmed with a modified Etest. Standardized inocula were prepared by diluting overnight cultures 1:35 (vol/vol) in sterile LB broth and incubating them at 37°C with shaking. When an optical density at 600 nm (OD_600_) of 0.6 was reached, a 1.5-ml volume was removed and centrifuged at 16,000 relative centrifugal force for 1 min. The supernatant was discarded, and 7 µl of wet pellet was transferred into 5 ml of 0.9% sterile saline in a 15-ml glass tube. The suspensions were adjusted to a bacterial concentration equivalent to a 1.0 McFarland turbidity standard and inoculated onto LB agar with sterile polyester-tipped swabs. Plates were allowed to dry for 10 min prior to the application of CST Etest strips (BioMérieux). Sample plates were imaged after 20 h of incubation at 37°C. *K. pneumoniae* Etest results not presented here are included in Fig. S3.

10.1128/mBio.01549-17.3 FIG S3  *K. pneumoniae* CST susceptibilities. CST susceptibility testing of *K. pneumoniae* strains was done by modified Etest. CST concentrations are in µg/ml. Images are representative of two or three separate tests. Download FIG S3, TIF file, 1.6 MB. Copyright © 2017 Crawford et al. 2017 Crawford et al. This content is distributed under the terms of the Creative Commons Attribution 4.0 International license.

### Chemokine treatment.

Recombinant human CXCL9 and CXCL10 were purchased from PeproTech (Rocky Hill, NJ) and reconstituted at 1 mg/ml in distilled water containing 0.3% human serum albumin. Overnight bacterial cultures were used to inoculate sterile LB medium at an OD_600_ of 0.1 to 0.2. Subcultures were incubated at 37°C with continuous shaking until an OD_600_ of 0.6 was attained. Bacteria were then diluted to a concentration of 3 × 10^6^ CFU/ml in 10 mM potassium phosphate buffer (pH 7.4) supplemented with 1% trypticase soy broth and treated with the chemokine indicated or an equal volume of 0.3% human serum albumin (untreated control) in the wells of a 96-cluster plate (12). After 2 h of incubation at 37°C, serial dilutions were prepared and inoculated onto LB agar plates that were incubated overnight at room temperature prior to colony enumeration.

### Lipid A isolation from whole cells.

Membrane lipids were extracted, and lipid A isolated, by an optimized small-scale hot ammonium isobutyrate-based protocol (37). Briefly, bacteria were streaked onto LB agar to obtain individual colonies. From these colonies, cultures were prepared in 5 ml of LB medium. Following overnight incubation at 37°C with continuous shaking, bacteria were harvested by centrifugation (4,000 relative centrifugal force for 10 min) and pellets were treated with a 5:3 mixture of 70% (vol/vol) isobutyric acid and 1 M ammonium hydroxide (400-µl total volume). Samples were then incubated at 100°C for 30 to 45 min. After centrifugation (2,000 relative centrifugal force for 15 min) to remove cell debris, the supernatants were transferred to fresh tubes, combined in a 1:1 ratio of distilled water, frozen on dry ice, and lyophilized overnight. The resulting dry pellets contained whole-cell extracts of membrane lipids.

### MALDI-TOF mass spectrometry.

Dry lipid extracts were washed twice with 1 ml of methanol and then resuspended in 200 µl of a 2:1:0.25 chloroform-methanol-water solvent mixture. Aliquots of 1 µl each of norharmane matrix (10 mg/ml in 2:1 chloroform-methanol) and then analyte were spotted directly onto stainless steel target plates in duplicate. Mass spectra were recorded in negative-ion mode with a Bruker Microflex LRF MALDI-TOF mass spectrometer (Bruker Daltonics, Billerica, MA) operated in reflectron mode. The instrument was equipped with a 337-nm nitrogen laser, and analyses were performed at 39.5% global intensity. Typically, 900 laser shots were summed to acquire each spectrum and at least three mass spectra were acquired per spot. Electrospray tuning mix (Agilent, Palo Alto, CA) was used for mass calibration. Data were acquired and processed with flexControl and flexAnalysis version 3.4 (Bruker Daltonics Inc.). Mass spectra were smoothed by a Savitzky-Golay filter, and the baseline was corrected with TopHat. Molecular structures and *m/z* values of major ions identified in *E. coli* and *K. pneumoniae* lipid A mass spectra are presented in Fig. S1. *K. pneumoniae* lipid A mass spectra not presented here are in Fig. S2.

### Genetic complementation and molecular cloning.

Complementation of isolate MS84 with *mgrB* was accomplished as previously reported for genetic complementation in KKBO4 (24). Briefly, native *mgrB* and flanking regions were amplified from the genomic DNA of isolate BL12125 by high-fidelity PCR with primers AvaI_mgrB_F1 (5′-GACTAGCTCGGGAACACGTTTTGAAACAAGTCGATGATTC-3′) and EcoRI_mgrB_R1 (5′-CTAGTCGAATTCCACCACCTCAAAGAGAAGGCGTTC-3′). PCR mixtures were prepared with Phusion DNA polymerase and incubated at 98°C for 30 s prior to 32 cycles of 98°C for 10 s, 62°C for 15 s, and 72°C for 20 s, followed by a final extension at 72°C for 7 min. The *mgrB* amplicon was purified with the MinElute PCR purification kit (Qiagen, Hilden, Germany) and sequence verified. The *mgrB* insert was cloned into pACYC184 (New England Biolabs, Ipswich, MA) following double restriction digestion of each with AvaI and EcoRI-HF, and ligation with T4 DNA ligase. The resulting construct, pACYC::*mgrB*, was electroporated into *E. coli* DH5α. Following sequence validation, pACYC::*mgrB* was transformed into freshly prepared, electrocompetent MS84 cells (1.8 kV, 200 Ω, 25 µF). An empty-vector control strain was also generated by transformation.

The introduction of *mcr*-*1* from isolate FI-4531 into *E. coli* J53 (F^−^ *met pro* Azi^r^ [azide resistant]) was accomplished by conjugation at a donor/recipient ratio of 1:10 (38). Bacteria were incubated at 35°C for 16 h, and transconjugants were selected on Mueller-Hinton agar supplemented with CST (2 µg/ml) and sodium azide (150 µg/ml). Transfer of the *mcr*-*1* resistance determinant was confirmed by PCR. For *mcr*-*1* expression by *K. pneumoniae* ATCC 13883, the *mcr*-*1* gene and its native promoter were amplified by PCR and cloned into pBCSK. The generation of ATCC 13883 harboring pBCSK::*mcr*-*1* or the empty vector alone has been described previously (15).

### Examination of chromosomal loci associated with CST resistance.

PCR amplification of chromosomal loci was performed by using primer pairs and cycling conditions previously reported for *phoP*, *phoQ*, *pmrA*, and *pmrB* (21) or for *mgrB* (19). PCR amplicons were visualized by gel electrophoresis, and the nucleotide sequences determined were compared to those of *K. pneumoniae* reference strains of the same sequence type to identify mutations/disruptions. The status of *phoP*/*phoQ*, *pmrA*/*pmrB*, and *mgrB* maintained by the 10 MDR *K. pneumoniae* isolates from Pakistan was also examined by using available draft whole-genome sequences available under accession numbers MAGC00000000 and MAGE00000000-MAGM00000000 (20). The genome of *K. pneumoniae* HS11286 was used for comparison (39). Where appropriate, the absence of *mcr* variants was confirmed on assembled genomes with CLC Genomics Workbench 9.5.4 by using the reference sequences NG_050417.1 (*mcr*-*1*), NG_051171.1 (*mcr*-2), and KX236309 (*mcr*-*1.2*). Insertion sequences were identified with ISfinder (40). For *K. pneumoniae* isolate BL849, no *mgrB* sequence or flanking regions could be identified by either *in silico* PCR or read mapping.

### Statistical analysis.

Statistical analysis and graphing were performed with GraphPad Prism 7 software; a *P* value of <0.05 was considered significant. Statistically significant differences among treatment groups were determined by one-way analysis of variance with Bonferroni’s multiple-comparison test or, when only two groups were experimentally assayed, an unpaired Student's *t* test. The EC_50_ of CXCL10-mediated bactericidal activity was determined by using the sigmoidal dose-response equation of nonlinear regression and is presented as the EC_50_ and the 95% confidence interval.
