# Supplementary figures and images for: CXC Chemokines Exhibit Bactericidal Activity against Multidrug-Resistant Gram-Negative Pathogens
Source: mBio. 2017 Nov 14;8(6):e01549-17. doi: 10.1128/mBio.01549-17 (PMC5686536; doi:10.1128/mBio.01549-17)

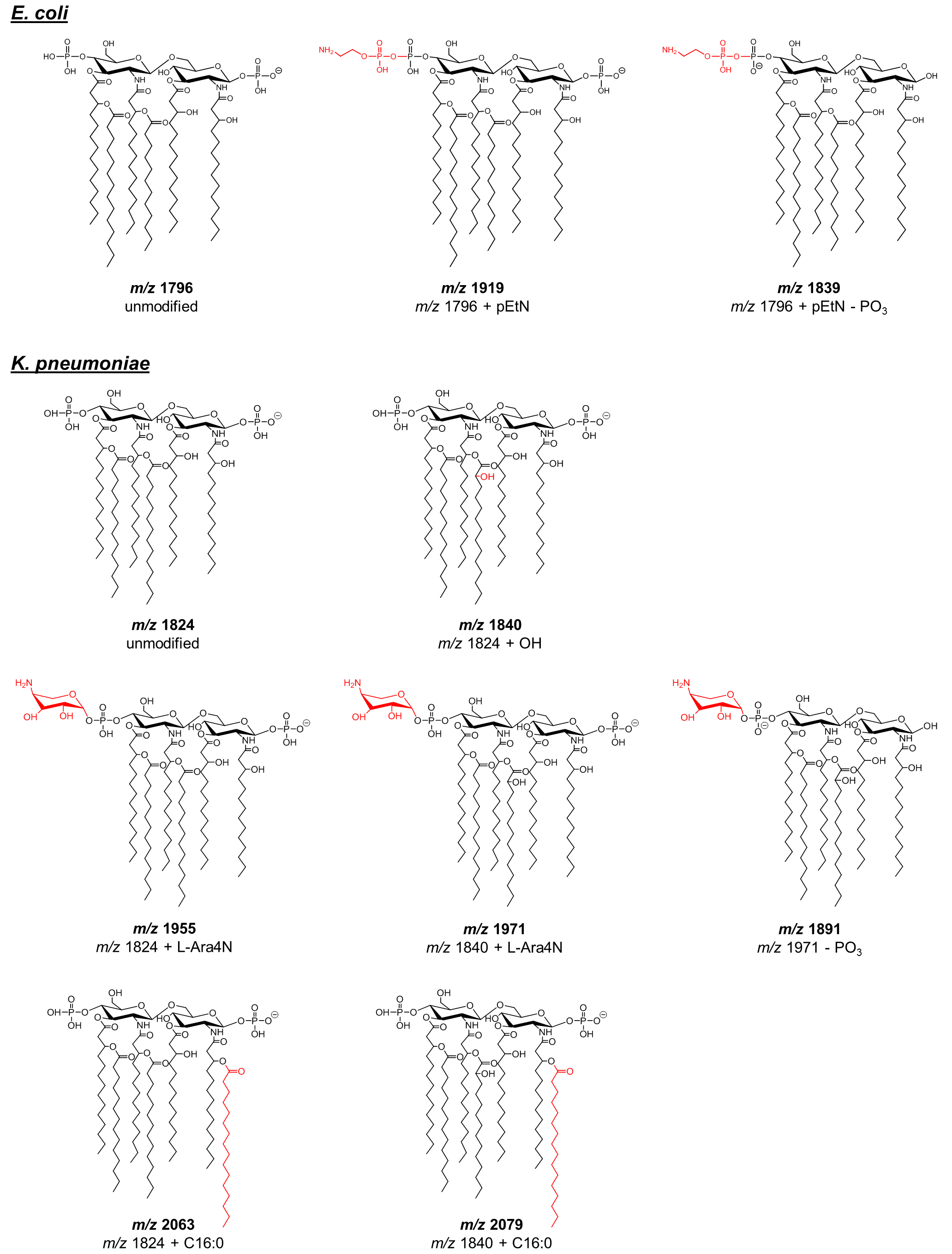

Supplement: FIG S1 [file mbo006173585sf1.tif]

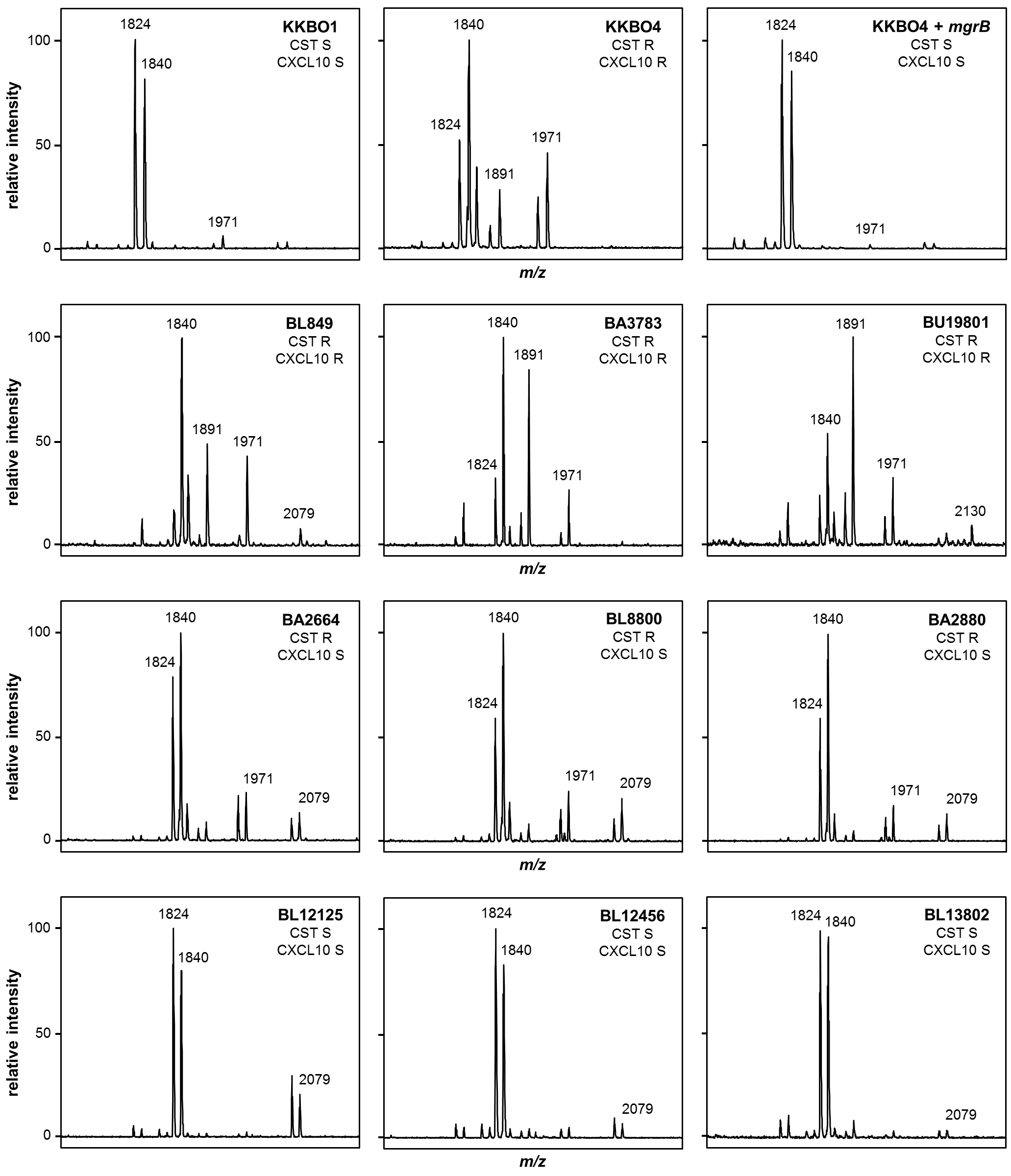

Supplement: FIG S2 [file mbo006173585sf2.tif]

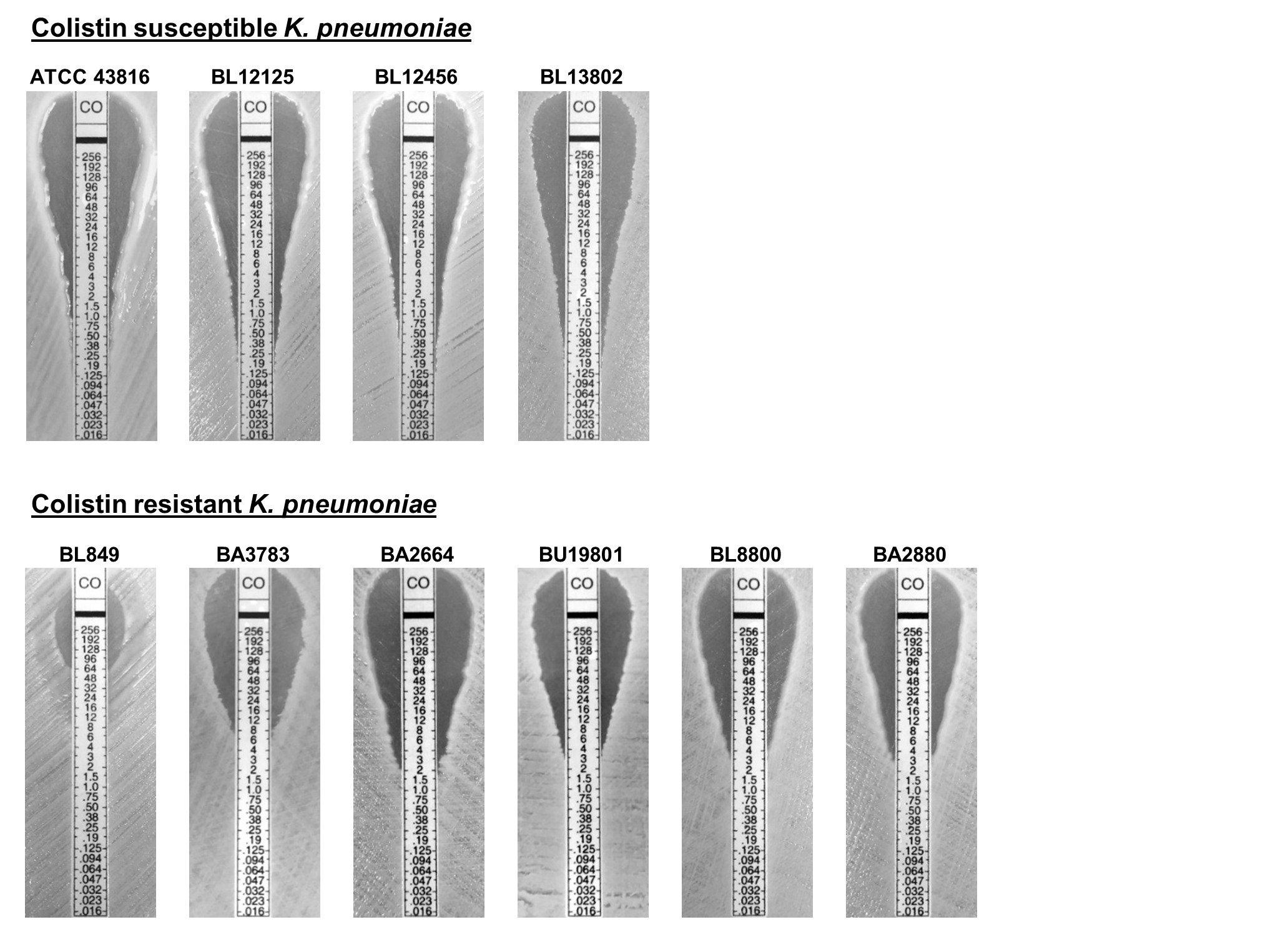

Supplement: FIG S3 [file mbo006173585sf3.tif]
